# Concomitant Associations between Lifestyle Characteristics and Physical Activity Status in Children and Adolescents

**Published:** 2019-03-10

**Authors:** Konstantinos D Tambalis, Demosthenes B Panagiotakos, Glyceria Psarra, Labros S Sidossis

**Affiliations:** ^1^ Department of Nutrition and Dietetics, School of Health Science & Education, Harokopio University, Athens, Greece; ^2^ Department of Kinesiology and Health, Rutgers University, New Brunswick, NJ 08901, USA

**Keywords:** Physical activity, Feeding behavior, Children, Adolescents, Lifestyle

## Abstract

**Background:** To examine the concomitant associations between physical activities (PA) and lifestyle factors in a representative sample of children and adolescents.

**Study design:** Cross-sectional, observational study.

**Methods:** Population data were derived from a school-based health survey carried out in 2015 on 177,091 (51% boys) Greek children aged 8 to 17 yr old. PA, sedentary activities and sleeping habits were assessed through self-completed questionnaires. Dietary habits were evaluated using the Mediterranean Diet Quality Index for children and adolescents. Anthropometric and physical fitness measurements were obtained by trained investigators. Logistic regression models were estimated and adjusted for relevant covariates.

**Results:** More boys as compared to girls (65.1% vs. 50.7%, *P*<0.001) and children than adolescents (59.8% vs. 52.8%, *P*<0.001) met the recommendations for PA. Frequent fast food consumption and skipping breakfast were associated with inadequate PA levels. In the whole population, sufficient dietary habits, sufficient (>8-9 h/d) sleeping and accepted screen time increased the participant’s odds of adequate PA levels by 38% (95% CI: 1.32, 1.44), 5% (95% CI: 1.01, 1.09) and 21% (95% CI: 1.16, 1.26), respectively, while, overweight/obese and central obesity decreased the odds of adequate PA levels by 7% and 5%, respectively, after adjusting for several covariates. Participants with combination of healthy aerobic fitness/dietary habits/screen time had 60% increased odds for adequate PA levels than those with unhealthy choices.

**Conclusion:** Healthy aerobic fitness, dietary habits and screen time were strongly associated with PA status among children. The results support the development of interventions to help children adopt a healthy lifestyle.

## Introduction

Health, quality of life and physical activity (PA) are strongly interrelated^1^. Chronic disease factors, such as a sedentary lifestyle, are present not only in adults but also in children and adolescentsas well^2^. Increasing the incidence of these diseases is directly linked to lifestyle changes, specifically PA behaviors, eating habits and smoking^3^. Less physically active children are more expected to possess risk factors for cardiometabolic disease such as a lower level of HDL-C, higher blood pressure, increased serum insulin levels and excess fat^1,4,5^. Therefore, it is essential to promote PA programs early in life to prevent such diseases. Moderate-to-vigorous physical activity (MVPA) is essential for disease prevention and health promotion^5^. Children and adolescents should spend a minimum of 60 min per day in MVPA^6^.

Lifestyle factors could interact with each other in an antagonistic or synergistic way to influence PA status, so it is important to investigate the association of PA with several potentially modifiable lifestyle factors (such as dietary habits, sedentary activities, sleeping status, physical fitness, and obesity status) considered simultaneously^1,7-23^. Even though, several studies have explored the association of sleeping hours^7-9^, screen time^10-12^, body mass index (BMI) status^13-15^, physical fitness (PF)^16-18^ and dietary habits^19-23^ with PA among schoolchildren, only a few have included all these factors, simultaneously in multivariate models. Moreover, most studies have included a specific age range of participants; thus these results may not be accurate to the whole of children and adolescents, in both sexes. Specifically, several studies have observed no consistent associations between PA and sleep duration^7-9^, while, screen time was inversely associated with PA, among children^10-12^. Moreover, though PA is an accepted strategy in the treatment of established childhood obesity, the role of PA in the prevention of obesity is less apparent^12-15^. Additionally, numerous studies among schoolchildren have speculated that PA was positively and well correlated with aerobic fitness^16-18^ and directly associated with healthy dietary habits^19-23^.

Thus, we aimed to explore the association between PA status and several lifestyle characteristics taking into consideration numerous potential confounders using data from a large, population-representative study in Greek schoolchildren aged 8-17 yr old.

## Methods

### Participants

Population-based, representative data were derived from a nation-wide school-based health survey in Greece. Specifically, anthropometric, nutrition, PA, sedentary habits, and PF data along with information on age and sex were collected from Mar 2015 to May 2015. Overall, 177091 (51% boys and 49% girls) children aged 8-17 yr old agreed to participate in the study.

Parents were informed in writing for the purposes of this school health survey. Verbally informed consent for the child to participate in the measurements was obtained from Physical Education (PE) teachers.

As the measurements were included in an obligatory school curriculum, verbal informed consent by the students was considered sufficient. The working sample was representative of the entire Greek population. Participation rate was almost 40% of the total population (chi-square *P*-value as compared to the current sample with the age-sex distribution of all Greek areas = 0.93).

### Assessment of demographic and anthropometric data

Demographic information of students was obtained from each school headmaster. Children’s height, body weight, and waist circumference were measured in the morning, using a standardized procedure. BMI status (e.g. normal weight, overweight, obese) was classified using the International Obesity Task Force age- and gender-specific BMI cut-off criteria^24^. For the purposes of the present study, we compared normal-weight with overweight (including obese) participants. Central obesity was defined as waist circumference (cm) -to-height (cm) ratio (WHtR) ≥0.5^25^. PE professionals performed all anthropometric measurements.

### Assessment of physical fitness levels

The Euro-fit PF test battery was used to evaluate children's PF levels^26^. The battery consists of 5 tests, i.e. (a) a multi-stage 20 m shuttle run test (20 m SRT), to estimate aerobic performance; (b) a maximum 10×5 m shuttle run test (10×5 m SRT) to evaluate speed and agility; (c) a sit-ups test in 30 sec (SUs), to measure the endurance of the abdominal and hip-flexor muscles; (d) a standing long jump (SLJ), to evaluate lower body explosive power; and (e) a sit and reach (SR) test to measure flexibility. Moreover, schoolchildren were classified into low and healthy cardiorespiratory fitness (CRF) levels, which proposed CRF cut points to avoid cardiovascular disease risk for boys and girls aged 8- to 18-yr, respectively^27^.

### Assessment of dietary habits

Participating in children’s dietary, PA and sedentary habits were recorded via an electronic questionnaire that was completed at school with the assistance of their teachers. Students’ dietary habits were assessed with the use of a valid and reliable questionnaire, namely KIDMED (Mediterranean Diet Quality Index for children and adolescents)^28^. This index contains 16 yes or no questions, including dietary habits that are in accordance with the principles of the MD dietary pattern and the general dietary guidelines for youth, and habits that undermine them. Questions denoting a negative connotation with respect to a high-quality diet are assigned a value of −1, while those with a positive aspect are assigned a value of +1. Thus, the total KIDMED score ranges from 0 to 12 and is classified into 3 levels: ≥8, suggesting an optimal adherence to the MD (sufficient dietary habits); 4–7, suggesting an average adherence to the MD and an improvement needed to adjust dietary intake to guidelines (relatively sufficient dietary habits); and ≤3, suggesting a low adherence to the MD and generally a low diet quality (insufficient dietary habits).

### Assessment of self-reported physical activity and sedentary time

Patterns of PA and sedentary time were also self-reported. The questionnaire applied has been previously validated and used in children in other large-scale epidemiological studies^29^. It includes simple closed-type questions regarding children’s type, frequency, time and intensity of participation in (i) school-related PA (including activity during PE classes; (ii) organized sports activities and (iii) PA during leisure time. The frequency of all reported activities was multiplied by the minutes of MVPA and then divided by 7 to obtain the mean daily time children engaged in MVPA. Children who participated in MVPA at least 60 min per day were considered as meeting the recommendations for PA. Daily time spent in sedentary activities (e.g. television viewing, use of Internet for non-study reasons, playing with computer or/and console games) was also calculated for each student (via multiplying the weekly frequency of participation with the duration per bout of participation in sedentary activities and then dividing by 7). Using the threshold of two hours per day, as per current guidelines, students were classified as sedentary or not, i.e., exceeding (>2 h/d) or not (≤2 h/d) the recommended daily time spent in sedentary activities^30^. Moreover, sleep time was assessed through self-reported recordings. We classified as meeting the recommendations of sufficient sleep those children (aged 6-12 yr old) who were sleeping at least nine hours per day and those adolescents (aged 13-17-yr-old) who were sleeping at least eight hours per day. Children and adolescents that were sleeping less than the recommended hours were classified as having insufficient sleep^31^.

### Ethical approval

Ethical approval for the health survey was granted by the Ethical Review Board of the Ministry of Education and the Ethical Review Committee of Harokopio University (decision No 37/20-02-2013).

### Data analysis

Descriptive statistics were expressed as mean ± standard deviation or frequency (percentages). The chi-square test evaluated associations between the categorical variables and the Student's *t*-test were applied to evaluate differences in mean values of normally distributed variables. In order to assess the potential effect of several dietary habits on the ‘adequate vs. inadequate’ PA level, binary logistic regression analysis was implemented and OR with the corresponding 95% CI were calculated, adjusted for confounders. Furthermore, aiming to assess the potential effect of several demographic and lifestyle factors on the PA level, hierarchical binary logistic regression analysis was implemented and OR with the corresponding 95% CI were calculated to obtain adjusted association of covariates while controlling for confounding. The Hosmer and Lemeshow's goodness-of-fit test was calculated in order to evaluate the model’s goodness-of-fit and residual analysis was implicated using the dBETA, the leverage, and Cook’s distance D statistics in order to identify outliers and influential observations. Finally, discriminant analysis was used to explore the strength of each component in relation to the outcome. All statistical analyses were performed using the SPSS ver. 23.0 software for Windows (Inc., Chicago, Il, USA). Statistical significance level from two-sided hypotheses was set at *P*<0.05.

## Results

Basic descriptive statistics of the total sample and by sex of participants are presented in Table 1. A greater proportion of boys compared to girls (65.1% vs. 50.7%, *P*<0.001) and of children, compared to adolescents (59.8% vs. 52.8%, *P*<0.001) meeting the current recommendations for PA.

**Table 1:** Characteristics of participants in the study aged 8 to 17-yr-old

**Continuous variables**	**Total (n=177,091)**		**Boys (n=87,803)**		**Girls (n=89,288)**		***P*** ** value**
	**Mean**	**SD**	**Mean**	**SD**	**Mean**	**SD**	
Age (yr)	9.88	2.8	9.91	2.8	9.84	2.8	0.001
Body Mass Index, (Kg/m^2^)	19.7	3.8	19.8	3.8	19.5	3.7	0.001
Waist circumference, (cm)	70.4	10.7	71.6	11.1	69.2	10.2	0.001
KIDMED score, (0, 12)	6.7	2.4	6.7	2.4	6.8	2.4	0.001
Physical activity, (h/wk)	9.4	5.5	10.4	5.9	8.4	5.2	0.001
Screen time, (h/wk)	8.6	8.5	9.3	8.8	7.8	7.8	0.001
Sleeping time, (h/d)	8.6	1.6	8.6	1.6	8.6	1.6	0.565
20m shuttle run, (laps)	31.1	18.6	36.2	20.6	25.4	13.9	0.001
Standing long jump, (cm)	117.0	55.7	124.0	59.3	110.0	50.5	0.001
Sit and reach, (cm)	15.4	8.3	13.2	7.6	17.7	8.3	0.001
Sit-Ups in 30 seconds, (n/30s)	19.7	5.7	20.6	5.8	18.7	5.3	0.001
10X5-meter shuttle run, (sec)	21.5	3.4	21.0	3.4	22.1	3.3	0.001
**Dichotomous variables**	**Number**	**Percent**	**Number**	**Percent**	**Number**	**Percent**	***P*** ** value**
Children (8-12 yr)	128,134	72.5	65,161	50.9	62,973	49.1	0.001
Adolescents (13-17 yr)	48,975	27.5	25,660	52.5	23,315	47.5	0.001
Achieved 60 min/day MVPA	102,632	58.0	57,130	65.1	42,294	50.7	0.001

*P* values for differences between boys and girls; MVPA: Moderate to vigorous physical activity (current recommendations for PA)

Table 2 provides a description of the study participants by PA level (adequate vs. inadequate). Participants from both sexes and age ranges who classified with inadequate PA levels reported poorer dietary habits, increased screen time, sleeping less and having worse PF and anthropometric measurements in comparison to participants with adequate levels of PA from the same sex and age range (all *P*-values<0.05).

**Table 2:** Anthropometric and behavioral characteristics (mean, SD) according to physical activity status (adequate vs. inadequate), in Greek boys and girls (aged 8 to 17-yr-old) of the study

**Variable**	**Adequate**				**Inadequate**				***P*** ** value**
	**Boys**		**Girls**		**Boys**		**Girls**		
**Children**	**Mean**	**SD**	**Mean**	**SD**	**Mean**	**SD**	**Mean**	**SD**	
Age, (yr)	10.0	1.2	10.3	1.2	10.3	1.2	10.1	1.2	0.001
Body mass index (Kg/m^2^)	18.9	3.5	18.8	3.5	19.2	3.6	19.0	3.5	0.001
Waist circumference (cm)	68.5	10.0	67.3	10.2	69.7	10.2	68.5	10.3	0.001
Waist to height ratio	0.3	0.5	0.5	0.6	0.3	0.6	0.5	0.6	0.016
KIDMED score	7.0	2.4	7.2	2.3	6.6	2.4	6.9	2.4	0.001
Physical activity (h/wk)	13.6	4.8	12.6	4.2	4.6	1.6	4.5	1.6	0.001
Screen time, (h/wk)	7.6	7.7	6.2	6.6	9.0	8.5	7.4	7.5	0.001
Sleeping time (h/day)	8.8	1.6	8.9	1.5	8.6	1.6	8.6	1.6	0.001
20m shuttle run (laps)	34.1	18.0	26.5	13.7	29.2	17.1	23.6	13.0	0.001
Standing long jump (cm)	121.2	52.6	112.3	47.6	114.9	50.7	106.8	45.9	0.001
Sit and reach (cm)	13.1	7.2	17.3	7.9	12.6	7.2	16.6	7.7	0.001
Sit-Ups in 30 sec (n)	21.0	5.5	18.9	5.3	18.9	5.6	17.8	5.3	0.001
10X5-meter shuttle run (sec)	21.2	3.3	22.0	3.3	21.8	3.3	22.5	3.3	0.001
**Adolescents**									
Age, (yr)	14.3	1.2	14.1	1.2	14.8	1.4	14.7	1.4	0.001
Body mass index (kg/m2)	21.5	3.9	21.1	3.6	21.9	3.9	21.4	3.8	0.001
Waist circumference (cm)	77.7	10.8	73.1	10.0	78.9	10.9	74.9	9.7	0.001
Waist to height ratio	0.4	0.6	0.5	0.6	0.4	0.6	0.5	0.6	0.012
KIDMED score	6.4	2.4	6.3	2.3	5.8	2.4	5.8	2.3	0.001
Physical activity (h/wk)	13.3	5.0	11.8	4.1	4.4	1.6	3.9	1.7	0.001
Screen time (h/wk)	11.4	9.8	10.6	9.4	11.7	9.8	11.2	9.6	0.001
Sleeping time (h/day)	8.2	1.5	8.1	1.5	7.8	1.7	7.6	1.5	0.001
20m shuttle run (laps)	50.1	23.9	31.7	13.7	46.3	23.8	27.9	14.6	0.001
Standing long jump (cm)	143.2	73.9	115.3	60.6	135.8	76.7	107.9	60.2	0.001
Sit and reach (cm)	14.2	8.5	20.3	9.1	14.1	8.7	19.1	8.9	0.001
Sit-Ups in 30 sec (n)	24.5	5.5	21.4	5.4	22.8	5.7	18.5	4.9	0.001
10X5-meter shuttle run (sec)	19.4	3.3	21.1	3.0	19.9	3.4	21.7	3.3	0.001

*P* values for differences between adequate and inadequate physical activity status from the same sex.

In unadjusted binary logistic regression, skipping breakfast and frequent fast food consumption decreased the odds of adequate PA levels, while, eating pasta or rice almost every day, consuming nuts regularly, eating a second fruit every day, eating pulses more than once weekly, using olive oil at home and eating two yogurts and/or cheese daily was associated with higher odds of meeting the recommendations for PA, in both sexes (Table 3, Model 1). After adjusting for several covariates (e.g. age, BMI, waist circumference and sleeping hours), the food habits previously reported, remained significantly associated with PA levels, in both sexes (Table 3, Model 2). Further adjustment for screen time did not change the results (Table 3, Model 3).

**Table 3:** Results from logistic regression models that used to evaluate the association of children’s (8 to 17-yr-old) dietary habits with physical activity status (inadequate vs. adequate)

**Predictors**	**Model 1**		**Model 2**		**Model 3**	
	**OR**	**95% CI**	**OR**	**95% CI**	**OR**	**95% CI**
**Boys**
Skips breakfast (no vs. yes)	0.90	0.84, 0.96	0.93	0.87, 0.99	0.89	0.85, 0.94
Has a second fruit every day (no vs. yes)	1.28	1.24, 1.32	1.28	1.24, 1.31	1.30	1.26, 1.34
Has fresh or cooked vegetables more than once a day (no vs. yes)	1.13	1.09, 1.17	1.14	1.10, 1.18	1.14	1.10, 1.18
Consumes fish at least 2–3/week (no vs. yes)	1.17	1.14, 1.21	1.17	1.14, 1.20	1.21	1.18, 1.25
Eats pulses >1/week (no vs. yes)	1.21	1.17, 1.25	1.20	1.16, 1.24	1.21	1.16, 1.26
Eats pasta/rice almost every day (no vs. yes)	1.10	1.07, 1.13	1.11	1.08, 1.15	1.11	1.07, 1.14
Fast food consumption (≤1/week vs. >1/week)	0.72	0.68, 0.76	0.76	0.70, 0.82	0.74	0.69, 0.79
Consumes nuts at least 2–3/week (no vs. yes)	1.22	1.19, 1.26	1.24	1.20, 1.28	1.25	1.21, 1.28
Uses olive oil at home (no vs. yes)	1.17	1.09, 1.26	1.15	1.07, 1.24	1.13	1.05, 1.22
Takes two yoghurts and/or some cheese (40 g) daily (no vs. yes)	1.13	1.09, 1.17	1.13	1.09, 1.17	1.13	1.09, 1.17
Takes sweets/candy several times every day (no vs. yes)	1.01	0.97, 1.05	0.96	0.92, 1.00	0.97	0.94, 1.00
** Girls**
Skips breakfast (no vs. yes)	0.98	0.94, 1.02	0.99	0.96, 1.02	0.97	0.93, 1.03
Has a second fruit every day (no vs. yes)	1.28	1.24, 1.32	1.24	1.20, 1.28	1.25	1.21, 1.29
Has fresh or cooked vegetables more than once a day (no vs. yes)	1.20	1.16, 1.24	1.19	1.15, 1.23	1.19	1.15, 1.22
Consumes fish at least 2–3/week (no vs. yes)	1.08	1.05, 1.11	1.07	1.04, 1.10	1.09	1.06, 1.12
Eats pulses >1/week (no vs. yes)	1.12	1.09, 1.16	1.13	1.10, 1.16	1.14	1.10, 1.17
Eats pasta/rice almost every day (no vs. yes)	1.10	1.07, 1.13	1.10	1.07, 1.14	1.10	1.07, 1.13
Fast food consumption(≤1/week vs. >1/week)	0.74	0.68, 0.80	0.78	0.73, 0.83	0.78	0.73, 0.84
Consumes nuts at least 2–3/week (no vs. yes)	1.19	1.16, 1.23	1.18	1.15, 1.21	1.19	1.15, 1.22
Uses olive oil at home (no vs. yes)	1.05	1.00, 1.10	1.02	0.94, 1.11	1.00	0.92, 1.10
Takes two yoghurts and/or some cheese (40 g/daily) (no vs. yes)	1.14	1.10, 1.18	1.14	1.10, 1.18	1.14	1.10, 1.18
Takes sweets/candy several times every day (no vs. yes)	0.96	0.90, 1.12	0.96	0.90, 1.12	0.97	0.92, 1.02

Model 1: Unadjusted; Model 2: Adjusted for age, BMI, waist circumference and sleeping hours; Model 3: Model 2 + screen time

Participants with inadequate PA levels had a worse lifestyle profile as compared to those with adequate PA levels, stepwise logistic regression analyses (4 Models) in both sexes was applied to explore the potential associations of several related factors on PA levels (inadequate vs. adequate). The initial analysis (Model 1) revealed that increase in the age (per one year) increased the odds of adequate PA levels by 1% in boys and decreased the correspondent odds by 7% in girls. Moreover, being overweight/obese or centrally obese reduced the odds of adequate PA levels, in both sexes (Table 4). When the KIDMED index and sleeping status were added in the analysis (Table 4, Model 2), previous results remained stable, while, sufficient dietary habits and sufficient (>8-9 h/d) sleeping status increased the odds of adequate PA levels, in both sexes. After additional adjustment for screen time (Model 3), the results revealed a favorable influence of accepted screen time on PA levels. Additionally, when PF measurements were included in the analysis (Model 4), the influence of previous factors did not alter significantly, while better PF measurements (with the exception of SR) were related to increased odds of adequate PA levels, in both sexes.

**Table 4:** Results from logistic regression models that used to evaluate the association of children’s (8 to 17-yr-old) characteristics with physical activity status (inadequate vs. adequate)

**Predictors**	**Model 1**		**Model 2**		**Model 3**		**Model 4**	
	**OR**	**95% CI**	**OR**	**95%CI**	**OR**	**95% CI**	**OR**	**95% CI**
**Boys**								
Age (per 1 year)	1.01	1.00, 1.01	1.01	1.01, 1.02	1.01	1.01, 1.02	1.01	1.00, 1.02
Weight group (normal weight vs. overweight/obese)	0.96	0.92, 1.00	0.96	0.92, 1.00	0.94	0.91, 0.98	0.94	0.92, 0.96
Central obesity (no vs. yes)	0.96	0.92, 0.99	0.96	0.92, 1.00	0.95	0.90, 1.00	0.95	0.91, 0.99
KIDMED index (insufficient vs. sufficient dietary habits)			1.46	1.39, 1.53	1.46	1.39, 1.52	1.46	1.40, 1.52
Sleeping hours (insufficient vs. sufficient)			1.04	1.00, 1.08	1.05	1.01, 1.09	1.05	1.01, 1.09
Screen time (increased vs. acceptable)					1.27	1.23, 1.31	1.26	1.20, 1.32
20m shuttle run (per 1 lap)							1.03	1.01, 1.05
Standing long jump (per 1 cm)							1.01	1.00, 1.01
Sit and reach (per 1 cm)							1.00	0.99, 1.02
Sit-Ups in 30 sec (per 1repetition)							1.01	1.00, 1.01
10X5-meter shuttle run (per 1sec)							0.99	0.99, 0.99
**Girls**								
Age (per 1 year)	0.93	0.90, 0.96	0.94	0.93, 0.94	0.94	0.93, 0.94	0.94	0.93, 0.94
Weight group (normal weight vs. overweight/ obese)	0.90	0.85, 0.95	0.95	0.91, 0.99	0.94	0.91, 0.98	0.93	0.91, 0.97
Central obesity (no vs. yes)	0.94	0.89, 0.99	0.95	0.92, 0.98	0.96	0.93, 0.99	0.96	0.93, 0.99
KIDMED index (sufficient vs. sufficient dietary habits)			1.40	1.34, 1.47	1.30	1.24, 1.36	1.30	1.24, 1.36
Sleeping hours (insufficient vs. sufficient)			1.04	1.00, 1.08	1.05	1.01, 1.09	1.05	1.01, 1.10
Screen time (increased vs. acceptable)					1.17	1.13, 1.21	1.16	1.12, 1.19
20m shuttle run (per 1 lap)							1.03	1.01, 1.05
Standing long jump (per 1 cm)							1.01	1.00, 1.02
Sit and reach (per 1 cm)							0.99	0.98, 1.01
Sit-Ups in 30 sec (per 1 repetition)							1.01	1.00, 1.01
10X5-meter shuttle run (per 1sec)							0.99	0.98, 1.00

Model 1: Age and BMI group and Central obesity; Model 2: Model 1 + KIDMED index and Sleeping hours and Physical activity levels; Model 3: Model 2 + Screen time; Model 4: Model 3 + Physical fitness measurements.

In the whole population, sufficient dietary habits, sufficient (>8-9 h/d) sleeping and accepted screen time increased the participant’s odds of adequate PA levels by 38% (95% CI: 1.32, 1.44), 5% (95% CI: 1.01, 1.09) and 21% (95% CI: 1.16, 1.26), respectively, while, overweight/obese and central obesity decreased the odds of adequate PA levels by 7% and 5%, respectively, after adjusting for several covariates. In addition, better results in PF measurements were related to higher probabilities of meeting the current recommendations of PA.

Discriminant analysis by sex was applied to assess whether the predictors could better distinguish participants with adequate from inadequate PA levels ones. Standardized function coefficients suggest that aerobic fitness (0.95); screen time (0.47), and dietary habits (0.36), contributes more to distinguishing those who met the PA recommendations from those with inadequate PA levels, in both sexes.

Finally, taking into account results from the discriminant analysis, we categorized participants according to the frequency of meeting the CRF, dietary habits, and screen time guidelines and for combinations of those recommendations. Healthy CRF level increased on participant’s odds of having adequate PA levels by almost 40% as compared to those with unhealthy CRF level, in both sexes (Table 5). Moreover, in comparison with participants with unhealthy CRF/inadequate diet, those with healthy CRF/adequate diet had 48% increased probabilities to meet the PA guidelines. Moreover, individuals with a combination of healthy CRF/adequate diet/adequate screen time had 60% lower risk of inadequate PA levels than those with a combination of unhealthy CRF/inadequate diet/inadequate screen time.

**Table 5:** Results from logistic regression models that used to evaluate the association of children’s (8 to 17-yr-old) recommended guidelines for cardiorespiratory fitness (CRF) levels, dietary habits and screen time with physical activity status (inadequate vs. adequate).

**Predictors**	**All**		**Boys**		**Girls**	
	**OR** ^a^	**95% CI**	**OR** ^a^	**95% CI**	**OR** ^a^	**95% CI**
CRF (unhealthy vs. healthy)	1.40	1.32, 1.48	1.48	1.40, 1.56	1.32	1.26, 1.38
CRF + Screen (unhealthy vs. healthy)	1.46	1.40, 1.53	1.48	1.41, 1.54	1.43	1.36, 1.49
CRF + Diet (unhealthy vs. healthy)	1.48	1.42, 1.55	1.50	1.43, 1.56	1.46	1.40, 1.52
CRF + Screen + Diet (unhealthy vs. healthy)	1.60	1.53, 1.67	1.63	1.59, 1.67	1.58	1.51, 1.65

^a^Adjusted for age, body mass index, waist circumference, and sleep time.

## Discussion

The current study reported concomitant associations between lifestyle characteristics and PA status in a representative sample of Greek children and adolescents, taking into account several covariates.

Our results revealed that 42% of the surveyed population failed to meet the PA recommendations. In the United States, only 25% of children met the 2008 PA Guidelines^32^, while most recent data showed that 24.8% of adolescents 12- to 15-yr-old of age reported daily consumption of 60 min of MVPA^33^. Furthermore, on average, only 20% of children in Organization for Economic Co-operation and Development countries participated in MVPA daily^34^.

In addition, we found that boys were more physically active as compared to girls and children than adolescents. Meeting the PA recommendations differs by age and gender, with males and younger children being more physically active than their female and older counterparts, independently of the measurement method of PA^33,35^.

The current study revealed that adequate PA level was inversely associated with the odds of skipping breakfast and frequent fast food consumption, still after adjustment for potential confounders. Moreover, compliance with the recommendations of Mediterranean diet was significantly related to PA levels. In line with previous results, among children, healthy dietary habits were favorably related to PA, in both sexes^19-21^. Moreover, more physically active girls had healthier dietary habits^22-24^.

Although childhood obesity is of great concern in Greece, PA levels did not seem to be a major contributor of total or central obesity in our study. Specifically, adequate PA levels decreased the odds of overweight/obese and central obesity by only 7% and 5%, respectively. Although the role of PA in the prevention of obesity is less obvious, there are enough scientific evidence to support the notion that lower PA levels are associated with a higher prevalence of obesity in children and adolescents^13-15^. Furthermore, in order to use PA as a tool to promote effective prevention strategies against obesity, a clearer understanding of the behavioral, psycho-social, and environmental factors that influence PA is needed, including the interactions between PA and other lifestyle factors such as sedentary time, sleeping and dietary habits^36^.

Participants of this study with increased screen time consumption (>2 h/d) had lower odds of adequate PA levels by almost 20%, in both sexes. A study in nationally representative sample of 15,143 US youth revealed that participants who engaged in less PA watched more TV per week^37^. Similarly, in a crossover study, decreasing sedentary behaviors was an effective strategy to increase PA^38^.

In addition, our findings suggest that sufficient sleep duration (>8-9 h/d) is associated with slightly higher probabilities (OR: 1.01, 1.09, *P*<0.001) of adequate PA levels, in both sexes. In line with our results, an epidemiological cohort study of 275 8-yr-old children showed that more physically active participant reported more and better sleep^39^. In contrary, a study among 65 Australian children aged 8- to 11-yr-old the authors did not observe significant associations between time total sleep time and MVPA^8^.

Our findings revealed significant trends that as performances of PF variables were better (with the exception of SRT), odds of adequate PA levels were increased. Moreover, the current results indicated that adequate PA levels increased the odds of having a healthy CRF by almost 40%, in both sexes. In line with previous findings, participants with high MVPA presented higher odds of having a high CRF in comparison with their peers with low MVPA^40^. Regular PA and enhanced PF constitute a healthy lifestyle and potentially these children were more likely to be more physically active.

Finally, the current study considered the concurrent influence of CRF, dietary habits, and screen time recommendations on PA levels. The main finding was that children meeting no risk factor recommendations had 60% decreased odds of adequate PA than those meeting all of the recommendations. These factors seem to exert a synergistic effect on the odds of PA levels when examined concurrently. These findings reveal the usefulness of these risk factor guidelines with respect to PA levels in addition to quantify the effect of failing to meet multiple cut points concurrently.

This study was performed in a wide age range and examined several covariates. In Greece, secondary and primary education is required and, consequently, we had the opportunity to study a huge part of the population aged 8- to 17-yr-old.

Limitations include methodological issues and the fact that prospective confounding factors, such as socio-economic status etc., that were likely connected to PA status, have not been evaluated. The record of PA was self-reported led to an overestimation of PA, and therefore the absolute PA levels should be interpreted with caution. Moreover, the record of dietary habits, sleeping time, and sedentary time was self-reported, therefore subject to desirable reporting bias. Nevertheless, participant responses were anonymous; as a result, they had no reason to misreport.

## Conclusion

Sufficient dietary habits, sleeping and screen time increased the participant’s odds of adequate PA levels, while, obesity decreased the odds of adequate PA levels, after adjusting for several covariates. Participants with combination of healthy aerobic fitness/dietary habits/screen time had significantly increased odds for adequate PA levels than those with unhealthy choices. The results support the development of interventions to help children adopt a healthy lifestyle, in general.

## Acknowledgements

This study was supported by the Hellenic Ministry of Education and Religious Affairs, Secretariat General of Sports, OPAP S.A., Nestlé Hellas S.A., and the Department of Nutrition and Dietetics Graduate Program, Harokopio University of Athens.

## Conflict of interest statement

The authors declared no conflict of interest.

## Funding

None.

## Highlights

- Lifestyle factors could interact with each other to influence physical activity (PA) status.
- More boys than girls and children than adolescents met adequate PA levels.
- Inadequate PA levels are dependably associated with unhealthy dietary patterns.
- PA levels are also associated with obesity, screen time, sleep duration, and physical fitness.
- Lifestyle factors could interact with each other in a synergistic way to influence PA status.
